# Transforming growth factor β1 signaling links extracellular matrix remodeling to intracellular lipogenesis upon physiological feeding events

**DOI:** 10.1016/j.jbc.2022.101748

**Published:** 2022-02-19

**Authors:** Shinichiro Toyoda, Jihoon Shin, Atsunori Fukuhara, Michio Otsuki, Iichiro Shimomura

**Affiliations:** 1 Department of Metabolic Medicine, Osaka University Graduate School of Medicine, Suita, Osaka, Japan; 2 Department of Diabetes Care Medicine, Osaka University Graduate School of Medicine, Suita, Osaka, Japan; 3 Department of Adipose Management, Osaka University Graduate School of Medicine, Suita, Osaka, Japan

**Keywords:** adipocyte, adipose tissue, feeding, TGF-β1, ECM remodeling, lipogenic pathway, ADAM, a disintegrin and metalloproteinase, DNL, *de novo* lipogenesis, ECM, extracellular matrix, MMP, matrix metalloproteinase, TGF, transforming growth factor, TGFBR, TGF-β receptor, TGFBR1, TGF-β receptor type 1, TGFBR2, TGF-β receptor type 2, TGFBRi, TGFBR inhibitor, SMAD2/3, SMAD family member 2/3, TBS-T, Tris-buffered saline with Tween-20, TG, triglyceride

## Abstract

Adipose tissue dynamically changes its mass in response to external nutritional status, which plays an important role in maintaining the lipid homeostasis. Physiologically, feeding events are associated with the expansion of adipose tissue, but little is known about the detailed molecular mechanisms of this expansion. Here, using comprehensive transcriptome analysis, we found that levels of transforming growth factor β1 (TGF-β1), a key regulator of extracellular matrix (ECM) remodeling, were increased in adipose tissue under feeding conditions and associated with the lipogenic pathway. In addition, TGF-β receptors are highly expressed in adipose tissue, and pharmacological inhibition of TGF-β1 reduced adipose tissue mass and caused ectopic lipid accumulation in the liver. This reduced fat mass was associated with decreased gene expression in ECM remodeling and lipogenesis. Furthermore, similar results were observed in the adipose tissue of SMAD family member 3 knockout mice or upon systemic TGF-β neutralization, with significant reductions in both ECM remodeling and lipogenesis-related genes. Mechanistically, we found that insulin-induced TGF-β1 and cell-autonomous action remodels the ECM of adipocytes, which controls the downstream focal adhesion kinase–AKT signaling cascades and enhances the lipogenic pathway. Of note, destruction of collagens or matrix metalloproteinase/a disintegrin and metalloprotease activities, critical components of ECM remodeling, blocked TGF-β1-mediated focal adhesion kinase–AKT signaling and the lipogenic pathway. Taken together, this study identifies a previously unknown lipogenic role of TGF-β1 by which adipocytes can expand to adapt to physiological feeding events.

Lipid storage is an important characteristic of living organisms, and proper regulation plays a critical role in energy homeostasis (1, 2, 3). Adipose tissue/adipocytes are specialized organs/cells that store energy in the form of triglycerides (TGs) and provide stored lipids in response to systemic nutritional demands (1, 2, 3, 4). Loss of adipocytes, called lipodystrophy, causes severe metabolic disorders with abnormal lipid homeostasis, leading to ectopic fat accumulation in the liver (5, 6, 7). However, appropriate expansion of adipocytes is advantageous for managing systemic lipid homeostasis and inhibits ectopic lipid deposition (8, 9, 10, 11, 12). The importance of adipocytes has been well established (13), but the physiological process of lipid storage has not yet been fully elucidated.

Feeding events induce insulin secretion from beta cells of pancreatic islets (14), and hormones act as important mediators of fat storage in adipocytes (15, 16). Insulin activity is associated with alterations in several intracellular signaling cascades (17), among which the AKT pathway induces the expression of downstream genes, such as SREBP1 (sterol regulatory element–binding transcription factor 1), ChREBP (carbohydrate-responsive element-binding protein), ACC (acetyl-CoA carboxylase), ACLY (ATP-citrate lyase), FASN (fatty acid synthase), and SCD (stearoyl-CoA desaturase) (1, 18, 19). These lipogenic molecules enhance enzymatic processes to convert dietary carbohydrates into fat, called *de novo* lipogenesis (DNL), and the energy source is accumulated in specialized intracellular structures and lipid droplets of adipocytes (1, 18, 19). Although lipogenic genes and their functions are well characterized, their regulatory mechanisms in adipocytes have not yet been fully explored.

For proper lipid accumulation, adipocytes not only require lipogenesis but also need to establish appropriate extracellular environments (9, 20). A three-dimensional network of extracellular proteins, called the extracellular matrix (ECM), surrounds the outside environment of adipocytes and supports cellular structures and biological functions (9, 20). Nutritional stimuli dynamically remodel the extracellular environment, adjusting to systemic energy states, and various structural and enzymatic proteins, such as collagens, fibronectin, matrix metalloproteinases (MMPs), a disintegrin and metalloproteinases (ADAMs), and tissue inhibitors of metalloproteinases, are involved in the process of ECM remodeling (9, 20, 21). Alterations in the extracellular environment affect intracellular events, in which the downstream signaling cascade involving focal adhesion kinase (FAK) plays key roles, mediating extracellular information in intracellular processes (22, 23, 24). The importance of the ECM and related remodeling factors has been well established, but the physiological regulation and detailed downstream actions in adipocytes have not been fully investigated.

Feeding-linked lipid storage is associated with diverse cellular processes of adipocytes, such as protein synthesis and secretion (2, 4). Adipocyte-derived bioactive cytokines or hormones, called adipocytokines, play important roles in the control of energy homeostasis in an endocrine manner or an autocrine/paracrine manner (2, 4). Leptin is the first characterized adipocytokine that is induced during the feeding cycle and controls food intake and energy use *via* endocrine action in hypothalamic neurons (5, 25, 26). Adiponectin is a multifunctional adipocytokine that exerts a protective role *via* the receptors AdipoRs or T-cadherin in many cells and organs (27, 28, 29, 30). Autocrine actions of adipocytokines, such as insulin-like growth factor 1, fibroblast growth factor 21, and stromal cell–derived factor 1, play important roles in a wide range of biological processes in adipocytes, including development, thermogenesis, and insulin sensitivity (31, 32, 33, 34). However, little is known about adipocytokines that control feeding-linked lipid storage of adipocytes in an autocrine/paracrine manner.

Here, by using publicly available transcriptome datasets, we analyzed feeding-related adipose signatures and found the enhancement of transforming growth factor β (TGF-β) expression and its association with the lipogenic pathway. TGF-β1 has been well established as a pathological fibrotic factor in obese adipose tissue, but little is known about the physiological role. In this study, we showed how adipose-derived TGF-β1 controls the feeding-linked lipogenic process of adipocytes *via* a series of *in silico*, *in vivo*, and *in vitro* experiments.

## Results

### The feeding-related lipogenic pathway is associated with TGF-β1 and the related ECM remodeling factors in adipose tissue

**Figure 1:**
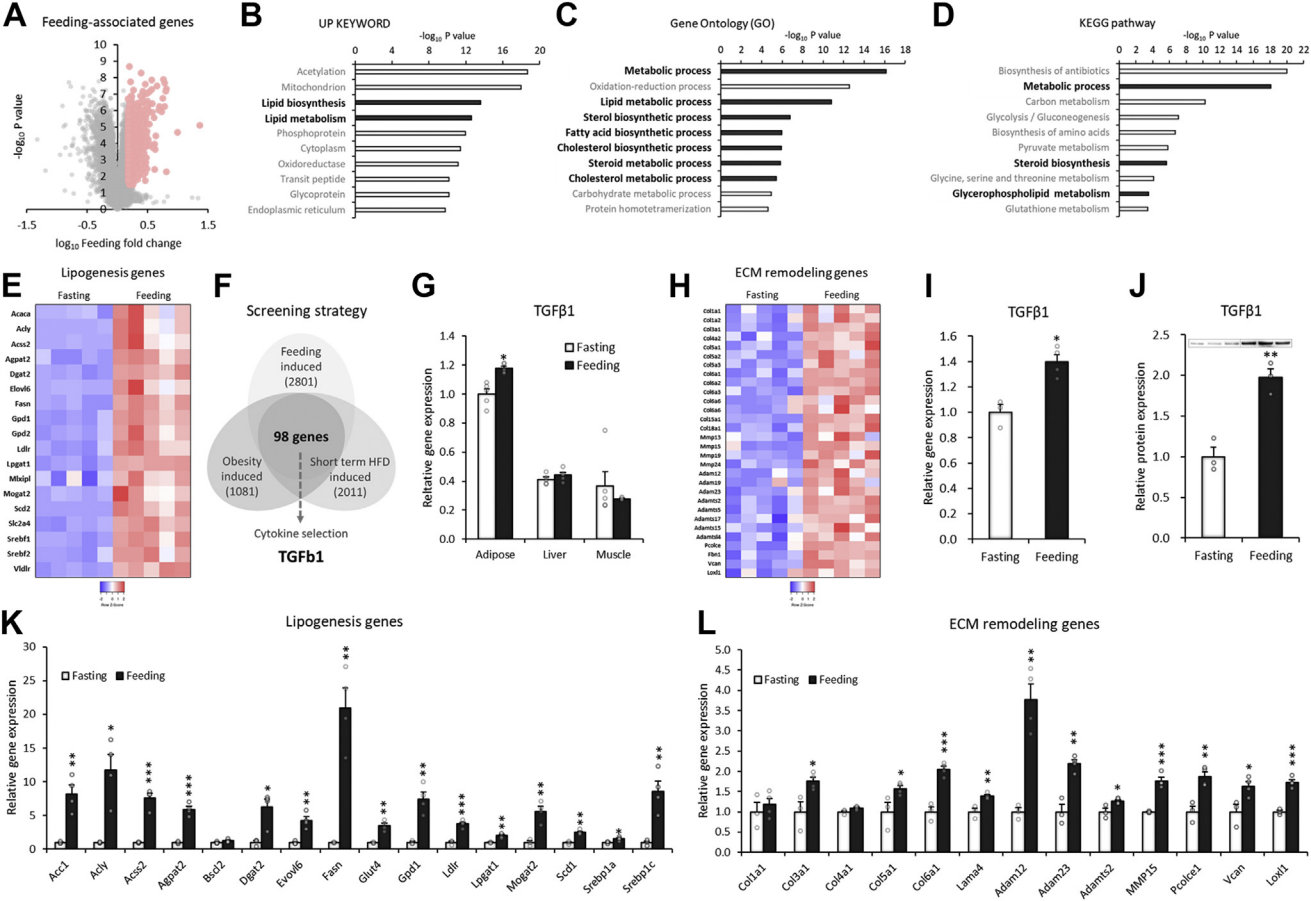
**The feeding-related lipogenic pathway is associated with TGF-β1 and the related ECM remodeling factors in adipose tissue.***A*–*E*, transcriptome analysis in adipose tissue of 24 h fasted C57BL/6J mice and chow-fed controls (GSE46495). Feeding-associated genes in mouse adipose tissues were selected (*A*) and analyzed with the Up Keyword (*B*), Gene Ontology (*C*), and KEGG pathway databases (*D*). *E*, heatmap of lipogenesis-related genes in mouse adipose tissues from 24 h fasting and *ad libitum* feeding states (GSE46495, n = 5). *F*, schematic diagram of microarray analysis to identify feeding-induced factors expressed in adipose tissue and adipocytes. The following Gene Expression Omnibus datasets were used for the analysis: feeding-induced genes in mouse adipose tissue (GSE46495, fold change >1.17, *p* < 0.05; 2801 genes), short-term high-fat diet–induced genes in mouse adipose tissue (GDS5824, fold change >1.25, *p* < 0.05; 2011 genes), and obesity-induced genes in human adipose tissue (GDS3602, fold change >1.25, *p* < 0.05; 1081 genes). *G*, relative expression of the TGF-β1 gene in mouse adipose tissues, liver, and skeletal muscle from 24 h fasting and *ad libitum* feeding states (GSE46495, n = 5). *H*, heatmap of ECM remodeling–related genes in mouse adipose tissues from 24 h fasting and *ad libitum* feeding states (GSE46495, n = 5). *I*, relative TGF-β1 RNA and protein expression of gonadal WAT from C57BL/6J mice fasted for 48 h and mice refed for 48 h (n = 3). *J*, relative TGF-β1 protein levels in gonadal WAT of C57BL/6J mice fasted for 24 h or refed for 12 h (n = 3). *K* and *L*, relative expression of the indicated genes in gonadal WAT from 48 h fasted or refed mice (n = 3). Data are presented as the mean ± SEM. ∗*p* < 0.05, ∗∗*p* < 0.01, ∗∗∗*p* < 0.001. ECM, extracellular matrix; KEGG, Kyoto Encyclopedia of Genes and Genomes; TGF-β1, transforming growth factor β1; WAT, white adipose tissue.

To elucidate feeding-associated adipose behaviors, we first analyzed the gene profiles of mouse adipose tissue. The feeding event altered various gene expression levels, and we used the significantly upregulated genes (feeding/fasting, fold >1.5, *p* < 0.05) for the next bioinformatic analyses (Fig. 1*A*). The feeding-induced genes included many associated with lipid biosynthesis and metabolism among the pathways (Fig. 1*B*). Gene Ontology and Kyoto Encyclopedia of Genes and Genomes pathway analysis showed a significant enhancement in the process of lipid biosynthesis and accumulation (Fig. 1, *C* and *D*). The gene expression of lipogenic genes, including ACC, ACLY, DGAT2 (diacylglycerol O-acyltransferase 2), and FASN, was substantially enhanced during feeding intervention (Fig. 1*E*).

To identify adipocytokines that potentially associate with feeding-linked lipogenic pathways of adipocytes, we crosscompared the feeding-induced genes with other types of feeding-related genes: (1) genes upregulated during feeding in mouse adipose tissue; (2) genes upregulated by short-term high-fat diet (3 days) in mouse adipocytes; and (3) genes upregulated in white adipose tissue of obese human subjects. The comparative analysis led to the identification of TGF-β1 as a potential adipocytokine (Fig. 1*F*). The induction of TGF-β1 specifically occurred in adipose tissue but not in liver or muscle (Fig. 1*G*). TGF-β1 is well known as a master regulator of ECM remodeling (35, 36, 37, 38), and feeding-induced TGF-β1 gene expression is associated with several ECM remodeling genes (Fig. 1*H*). We validated the screening results in a fasting/feeding mouse model. Feeding intervention significantly enhanced the gene and protein expression of TGF-β1 (Figs. 1, *I* and *J* and S1); TGF-β1 protein expression was significantly decreased by 24 h fasting and gradually increased over time after 16 h and 24 h of refeeding in white adipose tissue in align with the body weight changes (Fig. S2, *A* and *B*). The fat mass was significantly reduced by 24 h fasting and slightly recovered after 8, 16, and 24 h of refeeding but not to the level of *ad libitum* control (Fig. S2*C*). Feeding-related lipogenesis genes (Fig. 1*K*) were associated with ECM remodeling genes (Fig. 1*L*). These data indicated that TGF-β1 and the related ECM remodeling are somehow linked to the lipogenic pathway of adipose tissue.

### Pharmacological inhibition of the TGF-β receptor reduces the adipose tissue level

**Figure 2:**
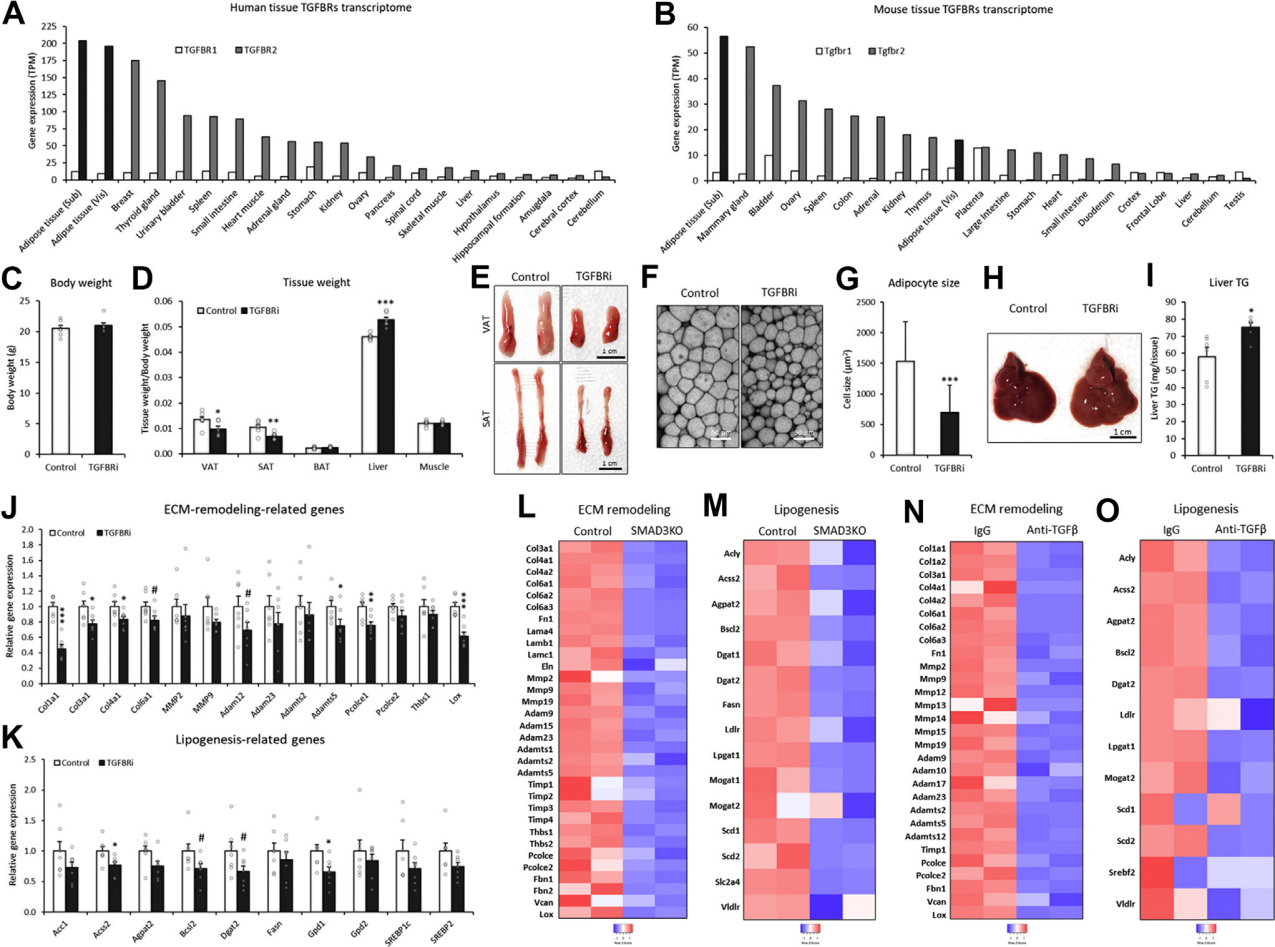
**Pharmacological inhibition of the TGF-β receptor reduces the adipose tissue level.***A* and *B*, gene expression of TGFBR1 and TGFBR2 in human and mouse tissues (human GTEx and mouse ENCODE transcriptomes). *C* and *D*, body and tissue weights of C57BL/6J mice 24 h after injection of TGFBR inhibitor (SB431542: 100 mg/kg BW) (n = 6). *E*, representative image of mouse adipose tissue 24 h after injection of SB431542. *F* and *G*, representative confocal image and cell size of visceral adipose tissue 24 h after injection of SB431542. *H*, representative image of the liver 24 h after injection of SB431542. *I*, triglyceride content of the liver 24 h after injection of SB431542 (n = 6). *J*, relative gene expression in visceral adipose tissues of mice injected with SB431542 (n = 6). *K*, relative gene expression in subcutaneous adipose tissues of mice injected with SB431542 (n = 6). *L* and *M*, heatmap of ECM remodeling and lipogenesis-related genes in visceral adipose tissue of SMAD3 knockout mice (GDS3985). *N* and *O*, heatmap of ECM remodeling and lipogenesis-related genes in visceral adipose tissue of the mice injected with TGF-β inhibitory antibody (GDS3985). Data are presented as the mean ± SEM. ^#^*p* < 0.1, ∗*p* < 0.05, ∗∗*p* < 0.01, ∗∗∗*p* < 0.001. BW, bodyweight; ECM, extracellular matrix; GTEx, genotype-tissue expression; SMAD3, SMAD family member 3; Sub- or SAT, subcutaneous white adipose tissue (inguinal WAT); TG, triglyceride; TGF-β, transforming growth factor β; TGFBR1, transforming growth factor β receptor type 1; TGFBR2, transforming growth factor β receptor type 2; Vis- or VAT, visceral white adipose tissue (gonadal WAT).

TGF-β1 mediates biological actions *via* the cell surface receptors TGF-β receptor type 1 (TGFBR1) and TGF-β receptor type 2 (TGFBR2). Binding of TGF-β1 to heterodimeric TGFBR1/TGFBR2 activates the downstream transcription factor SMAD family member 2/3 (SMAD2/3), which directly induces ECM remodeling–related gene expression (35, 36, 37, 38). These molecules were highly expressed in both human and mouse adipose tissues (Fig. 2, *A* and *B*). To understand *in vivo* action of TGF-β1 in adipose tissue, TGFBR inhibitor (TGFBRi; SB431542) was treated during fasting condition circumventing the influence of food intake and unwanted secondary/adverse effects. Pharmacological inhibition did not affect body weight (Fig. 2*C*) but strongly decreased the adipose tissue amounts (Fig. 2, *D* and *E*), which was associated with decreased adipocyte cell size (Fig. 2, *F* and *G*) and hepatic fat deposition (Fig. 2, *H* and *I*). Blockade significantly decreased the expression of ECM remodeling genes (Fig. 2*J*) and lipogenic genes (Fig. 2*K*). The treatment did not change gene expressions in lipolysis genes (Angptl4 [angiopoietin-like 4], ATGL [adipose triglyceride lipase], and HSL [hormone-sensitive lipase]) in the adipose tissue by TGFBRi treatment (Fig. S3*A*). Also, there were no significant differences in the serum level of free fatty acids between control and TGFBRi groups (Fig. S3*B*). These results have suggested that TGFBRi-reduced fat mass is more likely attributed to the lipogenesis pathway, rather than the lipolysis responses. Transcriptome analysis showed that knockout of SMAD3, a downstream transcription factor of TGF-β1, significantly downregulated both ECM remodeling–associated (Fig. 2*L*) and lipogenesis-associated genes (Fig. 2*M*) in adipose tissue. Similarly, neutralization of TGF-β activity by the blocking antibody decreased ECM remodeling–associated (Fig. 2*N*) and lipogenesis-related genes in adipose tissue (Fig. 2*O*). These data indicated that TGF-β1–TGFBR–SMAD3-mediated ECM remodeling was associated with the lipogenic pathway in adipose tissue.

### Cell-autonomous TGF-β1 activity controls the lipogenic pathway in adipocytes

**Figure 3:**
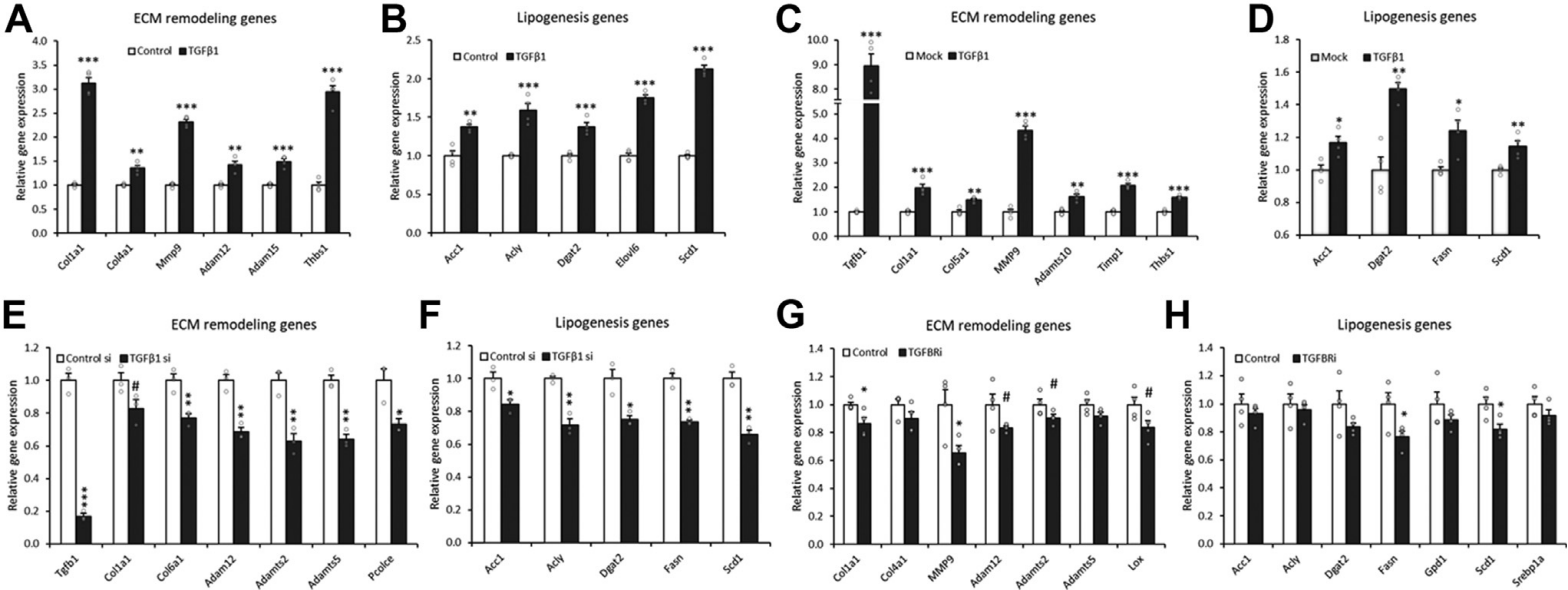
**Cell-autonomous TGF-β1 activity controls the lipogenic pathway in adipocytes.***A* and *B*, relative expression of the indicated genes after 12 h of TGF-β1 treatment in 3T3-L1 adipocytes (n = 4). *C* and *D*, relative expression of the indicated genes in 3T3-L1–tet–Tgfb1 adipocytes treated with 0.5 μg/ml doxycycline for 12 h (n = 4). *E* and *F*, relative expression of the indicated genes in 3T3-L1 adipocytes transfected with TGF-β1 siRNA (n = 3). *G* and *H*, relative expression of the indicated genes in 3T3-L1 adipocytes after 24 h of TGFBR inhibitor (SB431542: 10 μM) treatment (n = 4). Data are presented as the mean ± SEM. ^#^*p* < 0.1, ∗*p* < 0.05, ∗∗*p* < 0.01, ∗∗∗*p* < 0.001. TGF-β1, transforming growth factor β1; TGFBR, transforming growth factor β receptor.

Here, we hypothesized that direct and cell-autonomous TGF-β1 activity might control the lipogenic pathway in adipocytes. Treatment of 3T3-L1 adipocytes with TGF-β1 significantly upregulated ECM remodeling genes (Fig. 3*A*), which were associated with the gene expression of the lipogenic pathway (Fig. 3*B*). In a similar manner, doxycycline-mediated overexpression of TGF-β1 enhanced the gene expression of ECM remodeling (Fig. 3*C*) and lipogenic processes (Fig. 3*D*) in 3T3-L1 adipocytes. In contrast, knockdown of endogenous TGF-β1 by siRNA was enough to decrease the gene expression associated with ECM remodeling (Fig. 3*E*) and lipogenesis (Fig. 3*F*). Furthermore, blockade of the TGF-β1 receptor by the inhibitor in 3T3-L1 adipocytes, which inhibits the cell-autonomous activity of endogenous TGF-β1, also downregulated the expression of ECM remodeling–related (Fig. 3*G*) and lipogenesis-related genes (Fig. 3*H*). These data indicated that adipocyte-derived TGF-β1 regulates the lipogenic process in an autocrine/paracrine manner.

### TGF-β1-associated ECM and the downstream FAK–AKT signaling cascade control the lipogenic pathway in adipocytes

**Figure 4:**
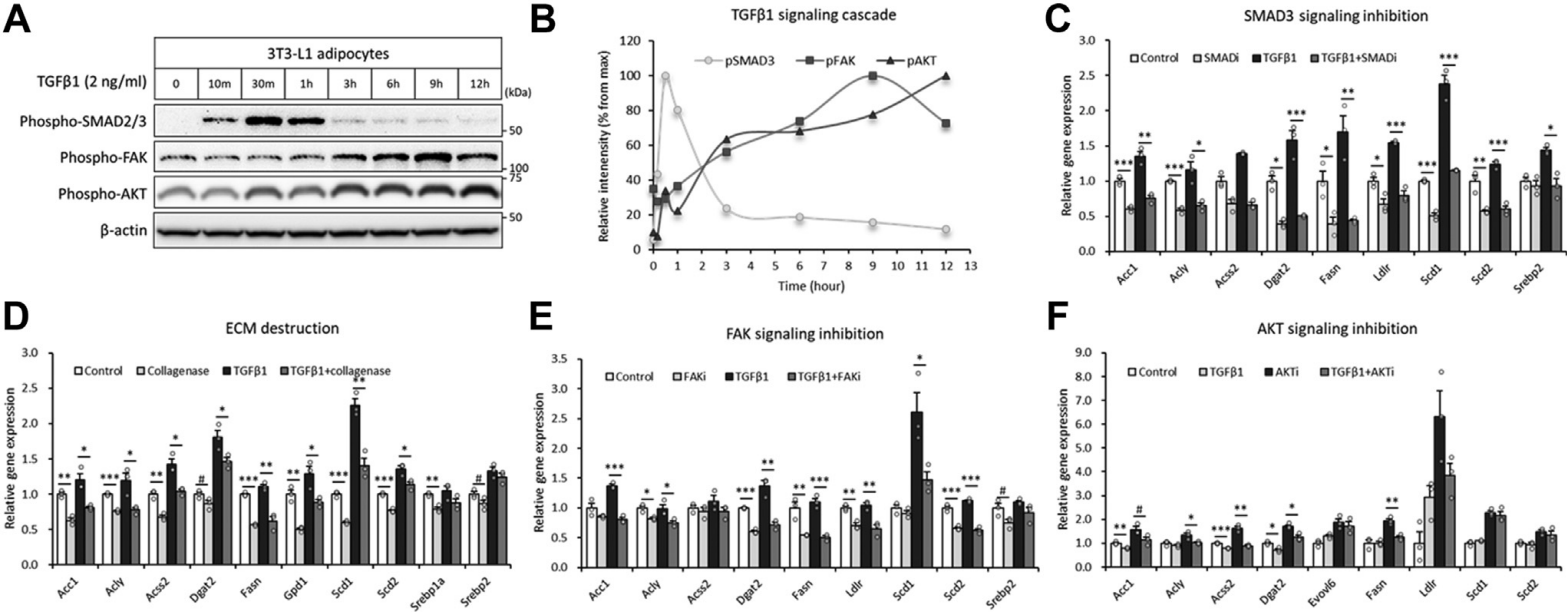
**TGF-β1-associated extracellular matrix and the downstream FAK–AKT signaling cascade control the lipogenesis pathway in adipocytes.***A* and *B*, phosphorylation levels of the indicated proteins in 3T3-L1 adipocytes treated with recombinant TGF-β1 (2 ng/ml). *C*, relative gene expression of lipogenesis-related genes after 12 h of TGF-β1 treatment with/without a SMAD3 inhibitor (SIS3 HCl: 10 μM) (n = 3). *D*, relative gene expression of lipogenesis-related genes after TGF-β1 treatment with/without collagenase (n = 3). *E*, relative gene expression of lipogenesis-related genes after TGF-β1 treatment with/without an FAK inhibitor (PF573228: 10 μM) (n = 3). *F*, relative gene expression of lipogenesis-related genes after TGF-β1 treatment with/without an AKT inhibitor (MK2206: 0.2 μM) (n = 4). Data are presented as the mean ± SEM. ^#^*p* < 0.1, ∗*p* < 0.05, ∗∗*p* < 0.01, ∗∗∗*p* < 0.001. FAK, focal adhesion kinase; SMAD3, SMAD family member 3; TGF-β1, transforming growth factor β1.

TGF-β1 is known to activate the SMAD3 transcription factor, which remodels the ECM with several ECM remodeling genes (35, 36, 37, 38), and extracellular alterations are associated with the intracellular FAK–AKT signaling cascade (22, 23, 24). To determine the molecular mechanism by which TGF-β1 regulates the lipogenic process in adipocytes, we first investigated the intracellular signaling effects. TGF-β1 treatment in 3T3-L1 adipocytes induced early phase phosphorylation of SMAD2/3, which peaked at 30 min, followed by the late-phase FAK–AKT cascade, which peaked at 9 and 12 h each (Fig. 4, *A* and *B*), indicating the time-course effects. The blockade of SMAD3 signaling by the specific inhibitor ablated TGF-β1-induced FAK–AKT axis (Fig. S4, *A*–*C*) and gene expression of the lipogenic pathway (Fig. 4*C*). Destruction of the extracellular collagen matrix by a low dose of collagenase, which did not affect adipocyte attachment and morphology, inhibited TGF-β1-induced lipogenic genes (Fig. 4*D*). Ablation of FAK signaling by the specific inhibitor blocked TGF-β1-mediated induction of lipogenesis-related genes (Fig. 4*E*). Blockade of AKT signaling abrogated TGF-β1-associated induction of lipogenesis-related genes (Fig. 4*F*). Of note, the inhibitors blocked both cell autonomous and TGF-β1-dependant lipogenesis genes (Fig. 4, *C*–*F*). These results are well aligned with the results in Figure 3, *C*–*F*, showing that ablations of cell autonomous TGF-β1–TGFBR activity by siRNA and TGFBRi without exogenous TGF-β1 treatment were enough to reduce lipogenesis gene expressions in adipocytes (Fig. 3, *C*, *D*, *G*, and *F*). Collectively, these data have suggested that adipose overexpressed and cell autonomous TGF-β1 controls lipogenesis genes by SMAD3–ECM–FAK–AKT axis.

### MMP–ADAM activity is critical for the TGF-β1-induced lipogenic process

**Figure 5:**
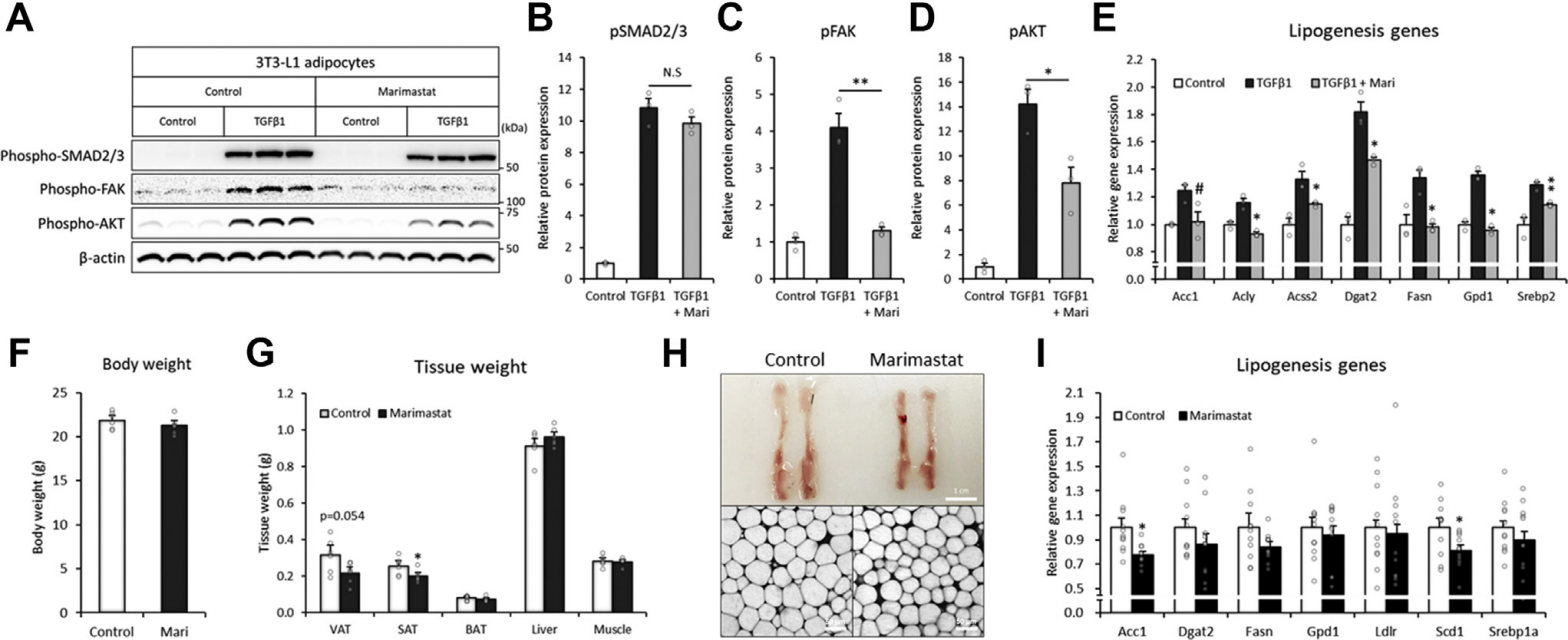
**MMP–ADAM activity is critical for the TGF-β1-induced lipogenic process.***A*–*D*, Western blot image of the indicated proteins in 3T3-L1 adipocytes after treatment with an MMP–ADAM inhibitor (marimastat: 20 μM) and/or recombinant TGF-β1 protein (2 ng/ml). *E*, relative expression levels of lipogenesis-related genes in 3T3-L1 adipocytes treated with recombinant TGF-β1 and 100 μM marimastat (n = 3). *F* and *G*, body and tissue weights of C57BL/6J mice 24 h after injection of 100 mg/kg BW marimastat (n = 6). *H*, representative confocal image of adipocytes 24 h after injection of marimastat. *I*, relative gene expression in gonadal adipose tissues of mice injected with marimastat (n = 11). Data are presented as the mean ± SEM. ^#^*p* < 0.1, ∗*p* < 0.05, ∗∗*p* < 0.01. ADAM, a disintegrin and metalloproteinase; BW, bodyweight; MMP, matrix metalloproteinase; TGF-β1, transforming growth factor β1.

ECM remodeling requires various enzymatic activities for the cleavage, maturation, or activation of ECM proteins, in which metalloproteinases, including MMPs and ADAMs, play major roles (24, 39, 40). As shown earlier, the expression levels were elevated in adipose tissue under feeding conditions (Fig. 1*L*) and associated with TGF-β1 and downstream actions (Fig. 2, *J*, *L*, and *M*). We next estimated whether the actions of these metalloproteinases are important for TGF-β1 signaling and the related lipogenic pathway. The ablation of metalloproteinase activity by marimastat, a pan-MMP and pan-ADAM inhibitor (41), did not affect TGF-β1-induced SMAD2/3 signaling but blocked the downstream FAK–AKT signaling cascade in 3T3-L1 adipocytes (Fig. 5, *A*–*D*). The inhibition also abrogated TGF-β1-induced lipogenic pathways in 3T3-L1 adipocytes (Fig. 5*E*). To understand the significance *in vivo*, we treated mice with the inhibitor. Pharmacological inhibition did not change the body weight in the *in vivo* mouse model (Fig. 5*F*) but reduced the fat mass and adipocyte size (Fig. 5, *G* and *H*), which was associated with decreased lipogenic pathways (Fig. 5*I*), phenocopying the loss of TGF-β1 activity (Fig. 2, *C*–*G*). These results suggested that TGF-β1-associated MMP–ADAM action, which is important for ECM remodeling, plays a crucial role in the downstream lipogenic process in adipocytes.

### Feeding-linked insulin activity directly upregulates TGF-β1 expression in adipocytes

**Figure 6:**
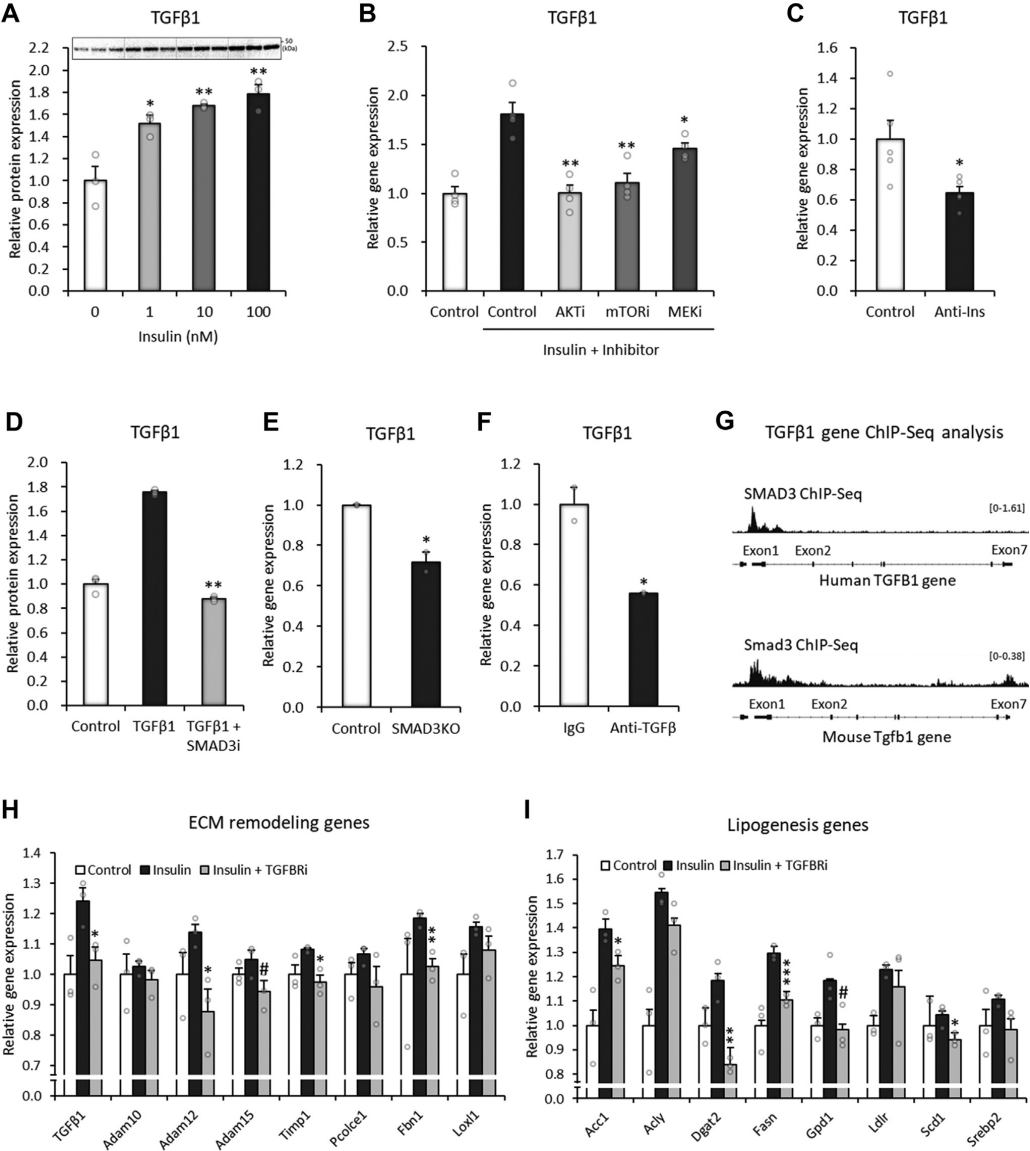
**Feeding-linked insulin activity directly upregulates TGF-β1 expression in adipocytes.***A*, Western blot image of TGF-β1 protein after 24 h of insulin treatment in 3T3-L1 adipocytes. *B*, relative gene expression of TGF-β1 in 3T3-L1 adipocytes after 48 h of insulin treatment with/without an AKT inhibitor (MK2206: 2 μM), mTOR inhibitor (rapamycin: 0.2 μM), or MEK inhibitor (U0126: 2 μM) (n = 4). *C*, relative gene expression of TGF-β1 in chicken adipose tissue in response to insulin neutralization (GSE35581, n = 4). *D*, relative gene expression of TGF-β1 in 3T3-L1 adipocytes treated with a SMAD3 inhibitor (SIS3 HCl: 10 μM) for 12 h with/without 2 ng/ml recombinant TGF-β1. *E* and *F*, transcriptome analysis of SMAD3 knockout mice (*E*) and TGF-β inhibitory antibody-injected mice (*F*) (GDS3985). *G*, SMAD3 ChIP-Seq peaks in human and mouse TGF-β1 gene regions (human SUM159 cells: GSM3736834; mouse embryonic stem cells: GSM3563733). The *y*-axes indicate RPM (reads per million mapped reads) units. *H*, relative gene expression of TGF-β1 and ECM remodeling genes in 3T3-L1 adipocytes after 18 h of TGFBR inhibitor treatment (SB431542: 10 μM), followed by 3 h of insulin responses (0.1 nM) (n = 3). *I*, relative gene expression of lipogenesis-related genes in 3T3-L1 adipocytes after 18 h of TGFBR inhibitor treatment (SB431542: 10 μM), followed by 3 h of insulin response (0.1 nM) (n = 3). Data are presented as the mean ± SEM. ^#^*p* < 0.1, ∗*p* < 0.05, ∗∗*p* < 0.01. ChIP-Seq, chromatin immunoprecipitation sequencing; ECM, extracellular matrix; MEK, MAPK/ERK kinase; mTOR, mammalian target of rapamycin; SMAD3, SMAD family member 3; TGF-β1, transforming growth factor β1; TGFBR, transforming growth factor β receptor.

The induction of TGF-β1 is a critical initial step for feeding-mediated lipogenesis in adipocytes. Feeding events are associated with increased circulating insulin levels, which affect various biological processes in target cells, including adipocytes (4). Here, we assumed the involvement of insulin in the regulation of TGF-β1 in adipocytes. Direct insulin treatment of 3T3-L1 adipocytes significantly enhanced the protein expression of TGF-β1 in a dose-dependent manner (Figs. 6*A* and S5). Ablations of insulin signaling molecules by specific inhibitors of AKT, mammalian target of rapamycin, and MAPK/ERK kinase blocked insulin-induced TGF-β gene expression (Fig. 6*B*). Transcriptome analysis showed that neutralization of insulin activity led to a substantial reduction in TGF-β1 gene expression in the *in vivo* adipose tissue of *Gallus gallus* (Fig. 6*C*), indicating insulin-mediated hormonal regulation both *in vivo* and *in vitro*.

During the course of this study, we found another regulatory mechanism that forms a positive feedback loop between TGF-β1 and SMAD3. Treatment of 3T3-L1 adipocytes with TGF-β1 substantially upregulated the gene expression of TGF-β1, which was blocked by an SMAD3 inhibitor (Fig. 6*D*). Transcriptome analyses showed that SMAD3 knockout or TGF-β neutralization reduced the gene expression of TGF-β1 in adipose tissue (Fig. 6, *E* and *F*). Chromatin immunoprecipitation sequencing analysis indicated that SMAD3 directly bound to TGF-β1 gene regions, especially around the exon 1 region, in both humans and mice (Fig. 6*G*). These data suggested that TGF-β1 is regulated by insulin-mediated hormonal action and the SMAD3-mediated positive feedback loop of TGF-β1 in adipocytes.

To further determine whether these two regulatory mechanisms for TGF-β1 are linked to each other, we added insulin with or without the inhibition of the TGF-β receptor in 3T3-L1 adipocytes. The insulin-mediated induction of TGF-β1 and ECM remodeling genes was strongly blocked by the inhibition of the TGF-β receptor (Fig. 6*H*). Insulin-associated lipogenic genes were attenuated by the inhibition of the TGF-β receptor (Fig. 6*I*), suggesting the importance of cell-autonomous TGF-β action in insulin activity. Collectively, two mechanisms for TGF-β1 regulation, insulin-mediated hormonal action and the SMAD3-mediated positive feedback loop, are not independent but highly coupled to regulate extracellular remodeling and lipogenic processes in adipocytes.

Finally, we investigated whether TGF-β1 has insulin-mimetic effects on insulin signaling, glucose uptake, and lipid accumulation. TGF-β1 pretreatment promoted not only the basal AKT phosphorylation (insulin: 0 min) but also the insulin-dependent AKT phosphorylation (insulin: 10, 30, 60, and 180 min) (Fig. S6*A*); whereas siRNA of endogenous TGF-β1 exhibited the opposite effects (Fig. S6*B*). TGF-β1 treatment alone increased 2-deoxyglucose uptake in 3T3-L1 adipocytes (Fig. S6*C*). In addition, long-term treatment of 3T3-L1 adipocytes with TGF-β1 for 48 h increased the lipid contents (Fig. S6*D*). These results have suggested that TGF-β1-induced AKT phosphorylation and glucose uptake are linked to the mechanistic process of DNL in adipocytes.

## Discussion

**Figure 7:**
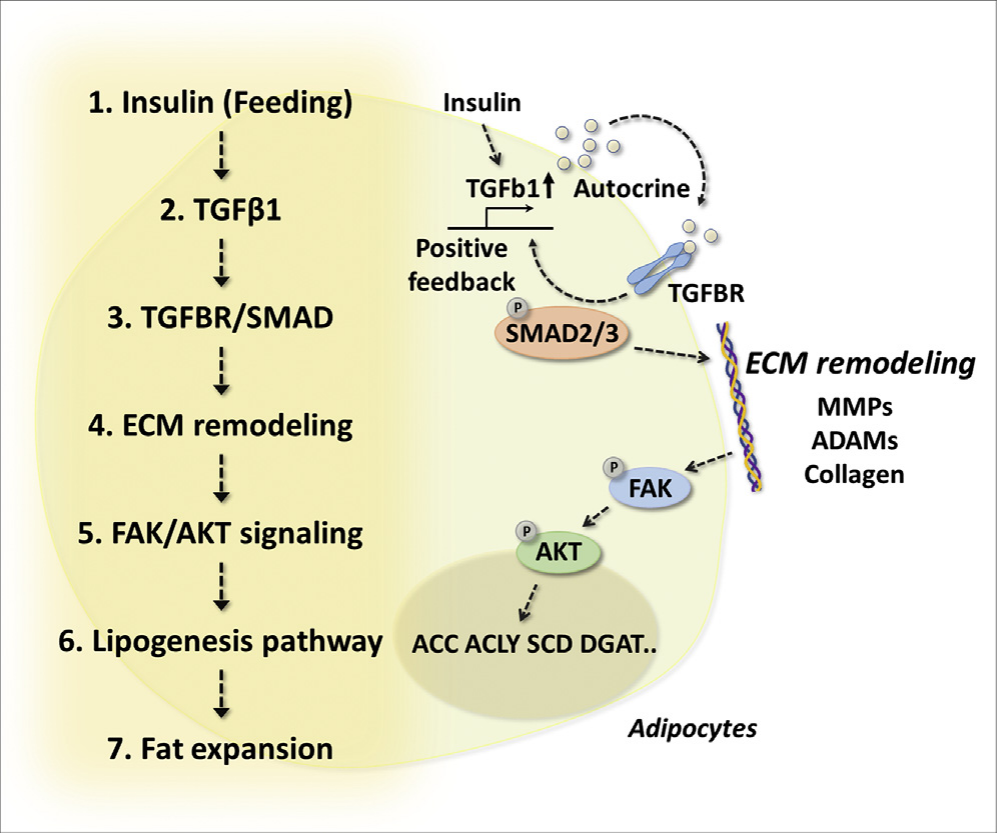
**Graphical summary.** Feeding-related insulin activity induces TGF-β1 expression in adipocytes. Autocrine action activates the TGFBR–SMAD3 axis, which even enhances the expression of TGF-β1 as a positive feedback loop. Moreover, it induces ECM remodeling with the induction of related genes such as collagen, MMPs, and ADAMs, which triggers intracellular FAK–AKT signaling and enhances lipogenesis. Cell-autonomous TGF-β1 activity plays an important role in linking the ECM to intracellular processes, which allows adipocytes to adapt to physiological feeding events and harmonizes extracellular and intracellular environments for lipid homeostasis. ADAM, a disintegrin and metalloproteinase; ECM, extracellular matrix; FAK, focal adhesion kinase; MMP, matrix metalloproteinase; SMAD3, SMAD family member 3; TGF-β1, transforming growth factor β1; TGFBR, transforming growth factor β receptor.

Physiologically, feeding events enhance lipid accumulation in adipocytes *via* DNL, but the detailed molecular basis has not been fully explored. Here, we showed the regulatory mechanism by which TGF-β1 regulates feeding-linked lipogenesis in adipocytes. Mechanistically, feeding events increase circulating insulin levels, which trigger TGF-β1 induction in adipocytes. Autocrine effects activate the TGFBR–SMAD3 pathway, which enhances the expression of TGF-β1 as a positive feedback loop and simultaneously increases ECM remodeling genes, including collagen, MMPs, and ADAMs. ECM remodeling induces intracellular FAK–AKT signaling, which is linked to the lipogenic process of adipocytes (Fig. 7).

Adipocytes dynamically remodel the extracellular composition and adjust the cellular size and lipid content according to the systemic nutritional states (9). However, the biological relationship between the ECM and lipogenesis has not been previously explored. In this study, we showed a significant linkage between the ECM and the intracellular lipogenic pathway, in which the FAK–AKT signaling cascade mediated the feeding-related adaptational process in adipocytes. TGF-β1 is well known as a profibrotic factor that induces deleterious fibrosis in a wide range of pathological processes (42, 43). Here, we identified a novel physiological role of TGF-β1 that links extracellular environments to the intracellular lipogenic process of adipocytes. The downstream transcription factor SMAD3 is known to regulate tissue remodeling in various pathophysiological conditions (35, 36, 37, 38). We showed the important involvement of TGF-β1 not only in ECM remodeling but also in feeding-linked lipogenesis of adipocytes. The remodeling of the extracellular environment involves various extracellular structural and enzymatic proteins, such as collagens, MMPs, and ADAMs, and defects associated with numerous developmental problems and pathological diseases (24, 39, 40). Importantly, we showed that the destruction of extracellular collagens or the inhibition of MMP–ADAM activity abolished TGF-β1-mediated ECM–FAK–AKT signaling and subsequent lipogenesis in adipocytes. Taken together, the results provide novel insight into how adipocytes adapt to feeding events and regulate lipogenesis, in which TGF-β1 and related ECM remodeling play key roles.

Lipid storage of adipocytes plays an important role in the regulation of systemic energy homeostasis (1, 2, 3, 4, 5, 6, 7). We showed that systemic inhibition of the TGF-β receptor caused significant loss of adipose tissue and ectopic fat deposition in the liver. These results indicated the importance of TGF-β1 not only in adipose lipogenesis but also in systemic lipid control. These findings are also well aligned with metabolic phenotypes of TGFb1 and TGFBR2 genes in genome-wide association studies that are associated with postprandial TG response (rs79134551, TGFBR2), high-density lipoprotein cholesterol level (rs15052, TGFb1), and type 2 diabetes (rs11466334, TGFb1), suggesting the significance in humans. In accordance with the results, previous studies have shown that defects in lipogenesis-related factors, including ACC, SCD, ACLY, DGAT1/2, and BSCL2 (Berardinell–Seip congenital lipodystrophy 2), lead to loss of adipose tissue and are associated with systemic metabolic abnormalities (44, 45, 46, 47, 48, 49, 50, 51). Similar phenotypes were found with defects of the ECM and related signaling molecules, including collagen, FBN, integrin, and FAK (52, 53, 54, 55, 56, 57).

Since the discovery of TGF-β in the 1970s (58), it has long been studied in a wide range of research areas. TGF-β includes three different isoforms (β1, β2, and β3) and has been shown to play various roles in adipocytes and adipose tissues under diverse conditions. TGF-β1 is known to inhibit the differentiation of preadipocytes to adipocytes *via* SMAD3-mediated transcriptional regulation (59, 60). Excessive TGF-β1 expression in adipose tissue under obese diabetic conditions is associated with fibrosis, inflammation, and mitochondrial dysfunction, which lead to insulin resistance (61, 62, 63). Adipose TGF-β1 is also involved in the sympathetic innervation of thermogenic adipose tissue (64). Exercise-induced TGF-β2 in subcutaneous adipose tissue contributes to systemic glucose tolerance and metabolism (65). TGF-β3 is likely to regulate the adipocyte number of subcutaneous fat (66). Knockout of TGFBR1 promoted beige adipogenesis and protected against high-fat diet–induced obesity (67). The biological actions of TGF-β seem likely to differ depending on the target cells, depots, conditions, or isoforms. It is important to determine what causes these functional differences, and further investigations need to be performed.

In the current study, we showed that TGF-β1 was regulated by two different mechanisms: feeding-associated insulin action and an SMAD3-mediated positive feedback loop. These two mechanisms are not separated but are more likely coupled to alter extracellular environments with intracellular processes for the lipogenic pathway in adipocytes (Fig. 6). Insulin-mediated intracellular signaling, especially the phosphorylation of AKT, occurs within 5 min, which is advantageous for inducing acute phase cellular uptake of glucose immediately after meals. In contrast, TGF-β1 induces gradual and progressive activation of AKT (Fig. 4, *A* and *B*), which might indicate its role in the adjustment of extracellular environments and the linked and prolonged lipogenic process.

Our findings shed new light on the physiological importance of TGF-β1, by which adipocytes synchronize the ECM with intracellular lipid homeostasis to adapt to feeding events. This is the first report to show the significant linkage between extracellular environments and intracellular functions of adipocytes, in this case lipogenesis. The importance of TGF-β1 in other research fields likely makes the system applicable to other cell types. We hope this study will help elucidate lipid homeostasis and could be a scientific basis for future drug development for metabolic diseases.

## Experimental procedures

### Cell culture and reagents

First, 3T3-L1 cells were differentiated into adipocytes using differentiation medium containing 3-isobutyl-1-methylxanthine (0.5 mmol/l), dexamethasone (1 μmol/l), insulin (1 μmol/l), and pioglitazone (10 μmol/l). The cells were used in experiments 7 days after differentiation. Treatment with recombinant human TGF-β1 (R&D Systems) was performed after changing the media to serum-free Dulbecco's modified Eagle's medium to avoid unknown serum effects. PlatE cells were used to produce retrovirus, followed by transfection in 3T3-L1 cells. Stable 3T3-L1 cells expressing tetracycline (tet)-inducible TGF-β1 (3T3-L1–tet–TGF-β1) or control cells (3T3-L1–tet–empty cell) were produced using the Retro-X Tet-On Advanced system according to the protocol supplied by the manufacturer (Clontech). The coding region of mouse TGF-β1 was subcloned into the expression vector pRetroX-Tight-Pur. Retroviral particles were generated using pRetroX-Tight-Pur-TGF-β1 or pRetroX-Tight-hygro-empty and pRetroX-tet-on advanced vectors. Infected 3T3-L1 cells were selected in 400 mg/ml G418 and 5 mg/ml puromycin. Other materials were as described: SB431542 (Selleck Chemicals), SIS3 HCl (Selleck), PF-573228 (Selleck), MK2206 (Selleck), collagenase (Sigma–Aldrich), rapamycin (Selleck), and U0126 (Selleck).

### siRNA

The differentiated 3T3-L1 adipocytes or mouse primary differentiated adipocytes (days 5–7) in dishes of 10 cm were treated with trypsin–EDTA and incubated at 37 °C for 2 min. The cells were washed in a 50 ml conical centrifuge tube and centrifuged at 500*g* for 5 min. Then, the siRNA mixture of Opti-MEM, siRNA solution (Qiagen; AllStars Negative Control siRNA and Flex tube siRNA), and RNAiMAX (Invitrogen) was prepared according to the instructions provided by the manufacturer. The cell pellet was gently resuspended in culture medium (1 × 10^6^ cells/ml), plated onto a 12-well dish with the siRNA mixture, and incubated for 2 days. At 2 days after siRNA treatment, the cells were maintained in serum-free medium for 18 h.

### Animal experiment design and method

Mice were housed in groups of one to three mice per cage, maintained in a room under controlled temperature (23 ± 1.5 °C) and humidity (45 ± 15%) on a 12 h dark/12 h light cycle, and had free access to water and chow (MF; Oriental Yeast). After intraperitoneal injection of 100 mg/kg/day SB431542 (Selleck) or 100 mg/kg/day marimastat (BB2516; Selleck), mice were fasted for 24 h or refed for 24 h and sacrificed for tissue samples; visceral adipose tissue (epididymal/gonadal adipose tissue), subcutaneous adipose tissue (inguinal white adipose tissue), and brown adipose tissue (intrascapular brown adipose tissue). All mouse studies were approved by the Ethics Review Committee for Animal Experimentation of Osaka University, Graduate School of Medicine, and performed in accordance with the Osaka University Institutional Animal Care and Use Committee Guidelines.

### RNA isolation and quantitative RT–PCR

Total RNA was isolated from tissues with TRI reagent (Sigma–Aldrich) using the procedure recommended by the manufacturer. A total of 500 ng of total RNA was reverse transcribed using Transcriptor Universal cDNA Master Mix (Roche). Quantitative RT–PCR analysis was performed using FastStart Essential DNA Green Master Mix (Roche) on a LightCycler 96 System. Relative gene expression was normalized to 36B4 or cyclophilin mRNA levels. The genes and corresponding primer sequences are listed in Table S1.

### Histology

Mouse adipose tissues were fixed with 4% paraformaldehyde/PBS. The tissue was blocked overnight at room temperature using PBS containing 0.1% Tween-20 and 10% goat serum. The tissue was incubated overnight at 4 °C with BODIPY 493/503 (1 μg/ml). After the samples were washed with PBS containing 0.1% Tween-20, the tissue was placed on a glass bottom 96-well plate with Fluoromount-G (SouthernBiotech) and dried at room temperature. Fluorescence images were recorded on an LSM710 confocal microscope (Zeiss). The BODIPY-positive area was measured as cell size.

### TG assay

The liver TG concentration was quantified using a commercially available colorimetric assay (Triglyceride Colorimetric Assay Kit; Cayman Chemical) according to the protocol supplied by the manufacturer.

### Glucose uptake assay

2-Deoxyglucose uptake kit (Cosmo Bio) was purchased, and analysis was performed according to the instructions provided by the manufacturer.

### Oil Red O staining

About 90 mg Oil Red O was dissolved in the 30 ml isopropanol and diluted with distilled 20 ml water. Differentiated 3T3-L1 adipocytes in 6-well plates were fixed in 4% paraformaldehyde for 1 h. After rinse with distilled water, the samples were stained with prepared Oil Red O working solution for 20 min at room temperature. The samples were washed with distilled water and 60% isopropanol. Oil Red O was eluted in 1 ml isopropanol per well. About 200 μl of the solution was dispensed per well in 96-well plate. The absorbance at 492 nm of each well was measured with a plate reader.

### Immunoblotting analysis

Cultured cells or tissue samples were lysed in lysis buffer (20 mmol/l Tris–HCl [pH 7.4], 1.0% Triton 3100, 150 mmol/l NaCl, 1 mmol/l EDTA, and 1 mmol/l EGTA) containing 1 mmol/l phenylmethylsulfonyl fluoride, 1.6 g/ml aprotinin, 10 g/ml leupeptin, and protease inhibitor cocktail (Nacalai Tesque, Inc). Protein concentration was determined by the bicinchoninic acid method (Pierce). The samples were used for Western blot analysis after concentrated sample buffer was added, and the samples were heated for 5 min at 95 °C. Equal amounts of protein were separated by SDS-PAGE and transferred electrophoretically to polyvinylidene difluoride membranes. The membranes were blocked for 1 h at room temperature using Tris-buffered saline (137 mmol/l NaCl, 20 mmol/l Tris–HCl, pH 7.6) containing 0.05% Tween-20 (TBS-T) and 5% skim milk. After three washes with TBS-T for 10 min each, the membranes were incubated overnight at 4 °C with primary antibodies against TGF-β1 (Abcam; catalog no.: ab179695), phospho-AKT (Ser473; Cell Signaling Technology; catalog no.: 4060), total AKT (Cell Signaling; catalog no.: 9272S), phospho-FAK (Tyr397; Cell Signaling Technology; catalog no.: 8556S), total FAK (Sigma–Aldrich; catalog no.: 05-537), phospho-Smad2/3 (Ser465/467, Ser423/425; Cell Signaling Technology; catalog no.: 8828), and β-actin (Sigma–Aldrich; catalog no.: 9062) in TBS-T and 5% skim milk. After three washes with TBS-T for 10 min each, the membranes were incubated for 1 h at room temperature with enhanced chemiluminescence horseradish peroxidase–linked secondary antibodies (GE Healthcare) in TBS-T and 5% skim milk. After extensive triple washing in TBS-T, the immunoreactive bands were visualized by Pierce Western Blotting Substrate Plus (Thermo Fisher Scientific) according to the manufacturers' protocols. Quantification was conducted by densitometry using ImageJ software (National Institutes of Health).

### Statistical analysis

All data are presented as the mean ± SEM. Differences between two groups were examined for statistical significance by Student’s *t* test. A *p* value <0.05 denoted the presence of a statistically significant difference.

## Data and resource availability

The datasets and source data generated during and/or analyzed during the current study are available from the corresponding author upon reasonable request.

## Supporting information

This article contains supporting information.

## Conflict of interest

The authors declare that they have no conflicts of interest with the contents of this article.
