# Supplementary material for: Transforming growth factor β1 signaling links extracellular matrix remodeling to intracellular lipogenesis upon physiological feeding events
Source: J Biol Chem. 2022 Feb 19;298(4):101748. doi: 10.1016/j.jbc.2022.101748 (PMC8931428; doi:10.1016/j.jbc.2022.101748)
Supplement: Supplemental Table S1 [file mmc1.docx]

**Supplemental table**

| **Table S1. Primers Used in Real-Time RT-PCR** | | |  |  |
| --- | --- | --- | --- | --- |
|  | **Forward Primer (5´→3´)** | **Reverse Primer (5´→3´)** | |  |
| 36B4 | GCTCCAAGCAGATGCAGCA | CCGGATGTGAGGCAGCAG | |  |
| Cyclophilin | CAGACGCCACTGTCGCTTT | TGTCTTTGGAACTTTGTCTGCAA | |  |
| Tgfb1 | ACCATGCCAACTTCTGTCTG | CGGGTTGTGTTGGTTGTAGA | |  |
| Col1a1 | GTGCTCCTGGTATTGCTGGT | GGCTCCTCGTTTTCCTTCTT | |  |
| Col3a1 | GGGTTTCCCTGGTCCTAAAG | CCTGGTTTCCCATTTTCTCC | |  |
| Col4a1 | GCCAAGTGTGCATGAGAAGA | AGCGGGGTGTGTTAGTTACG | |  |
| Col5a1 | CTCCAACACCTCCAATCCAG | GTCCTCCAATCCCCTCAAAG | |  |
| Col6a1 | GATGAGGGTGAAGTGGGAGA | CAGCACGAAGAGGATGTCAA | |  |
| Lama4 | TACTATGGAGACGCCAGGAC | TCATCGGTCAAGTCCCAGAC | |  |
| Adam12 | GTGTCTTCGGCGTTCACAAG | GAAACGGATTGCCTTGCTGT | |  |
| Adam23 | CTTTGGAGGCGTGTGTTCTC | TGGGACACCCCTGTTTCTTC | |  |
| Adamts2 | ACGTGGTGTCTTTGGAGACG | ACAGCATTGGTTTCTGGGTG | |  |
| Adamts5 | CACCTCCTCGCATCTTCACA | CCTGTTTCCATCCTGGCACT | |  |
| Mmp2 | CCCTCCCCCGATGCTGATAC | TCCGCCAAATAAACCGGTCCT | |  |
| Mmp9 | AGGGGCGTGTCTGGAGATTC | CCAGGGCACACCAGAGAACT | |  |
| Mmp15 | CCAAGCCCATCAGTGTCTGG | GGTTCCATCCGTAGGCGTTC | |  |
| Pcolce1 | TGAGTCGGATTACCCACCAG | ATATCGGCAGTACGTGTCAGG | |  |
| Pcolce2 | GAAGTGGCTTCATGGCAACG | CACGGGGTAATCTCGGTCTG | |  |
| Thbs1 | AACAACGAGGAGTGGACTGTAG | TCAGGAACTGTGGCGTTGG | |  |
| Vcan | TTGCTCATCGACGCACATGG | TTGTCATTGAGGCCGATCCAC | |  |
| Lox | AGTGAAGAACCAAGGGACATCG | CTTCAGCCACTCTCCTCTGTG | |  |
| Loxl1 | CCCATCTGTACTCCTTGCGATG | AGAAAGTCCGCTGTGCCTTG | |  |
| Acc1 | GAGGTACCGAAGTGGCATCC | GTGACCTGAGCGTGGGAGAA | |  |
| Acly | ACCCTTTCACTGGGGATCACA | GACAGGGATCAGGATTTCCTTG | |  |
| Acss2 | TCACGGGCAGGATTGATGAC | AAGTGCCGATTCCACCTCTG | |  |
| Agpat2 | TGGGCCTCATCATGTACCTTG | CGTTGCGTGTACCCTCTGG | |  |
| Bscl2 | GCTAAGAGTGGACGTGATCGG | GATGATTCGGCCACCTCTGG | |  |
| Dgat2 | GGCTACGTTGGCTGGTAACTT | CACTCCCATTCTTGGAGAGC | |  |
| Elovl6 | GAAAAGCAGTTCAACGAGAACG | AGATGCCGACCACCAAAGATA | |  |
| Fasn | GAGAAGCCATGTGGGGAAGATTTC | TGAGCAGGGACAGGACAAGAC | |  |
| Glut4 | GCGGATGCTATGGGTCCTTA | GTCCGGCCTCTGGTTTCAG | |  |
| Gpd1 | AGACCTCATCACGACCTGCT | CTCCAGCTGCTCAATGGACT | |  |
| Gpd2 | TTGAACAAAAATGGACAGGTTGA | CCAGAGACCCTTCCTTTCTGAA | |  |
| Ldlr | GAGGAACTGGCGGCTGAA | GTGCTGGATGGGGAGGTCT | |  |
| Lpgat1 | TTTAGGAATGGTGGCTTCCTGG | GGTTCACCAACATCACTGCTTC | |  |
| Mogat2 | CCCGTGTGTAGAGCCTCCTG | AGGTCTGTAACCTGCGCTCC | |  |
| Scd1 | TGGGTTGGCTGCTTGTG | GCGTGGGCAGGATGAAG | |  |
| Scd2 | GCATTTGGGAGCCTTGTACG | AGCCGTGCCTTGTATGTTCTG | |  |
| Srebp1a | ACTTTTCCTTAACGTGGG | GGAGAGTTGGCACCTG | |  |
| Srebp1c | GGAAGCTGTCGGGGTAGCG | ATGTGCAATCCATGGCTCCG | |  |
| Srebp2 | GTTGACCACGCTGAAG | ATATTGTGTGTTGTCCGC | |  |
| Angptl4 | GCATGGCTGCCTGTGGTAAC | ATCTTGCTGTTTTGAGCCTTGA | |  |
| Atgl | TGTGGCCTCATTCCTCCTAC | TCGTGGATGTTGGTGGAGCT | |  |
| Hsl | CCGCTGACTTCCTGCAAGAG | CTGGGTCTATGGCGAATCGG | |  |
