# Supplementary material for: Transforming growth factor β1 signaling links extracellular matrix remodeling to intracellular lipogenesis upon physiological feeding events
Source: J Biol Chem. 2022 Feb 19;298(4):101748. doi: 10.1016/j.jbc.2022.101748 (PMC8931428; doi:10.1016/j.jbc.2022.101748)
Supplement: Supplemental Figures S1–S6 [file mmc2.docx]

**
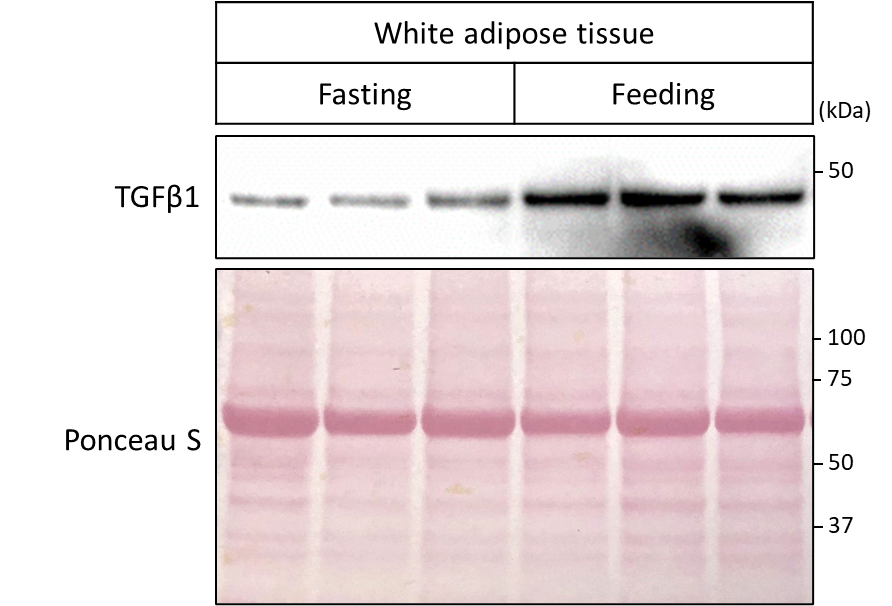
**

**Supplemental Figure 1.** Western blot image of TGFβ1 in gonadal WAT of C57BL/6J mice fasted for 24 hours or refed for 12 hours (n=3).


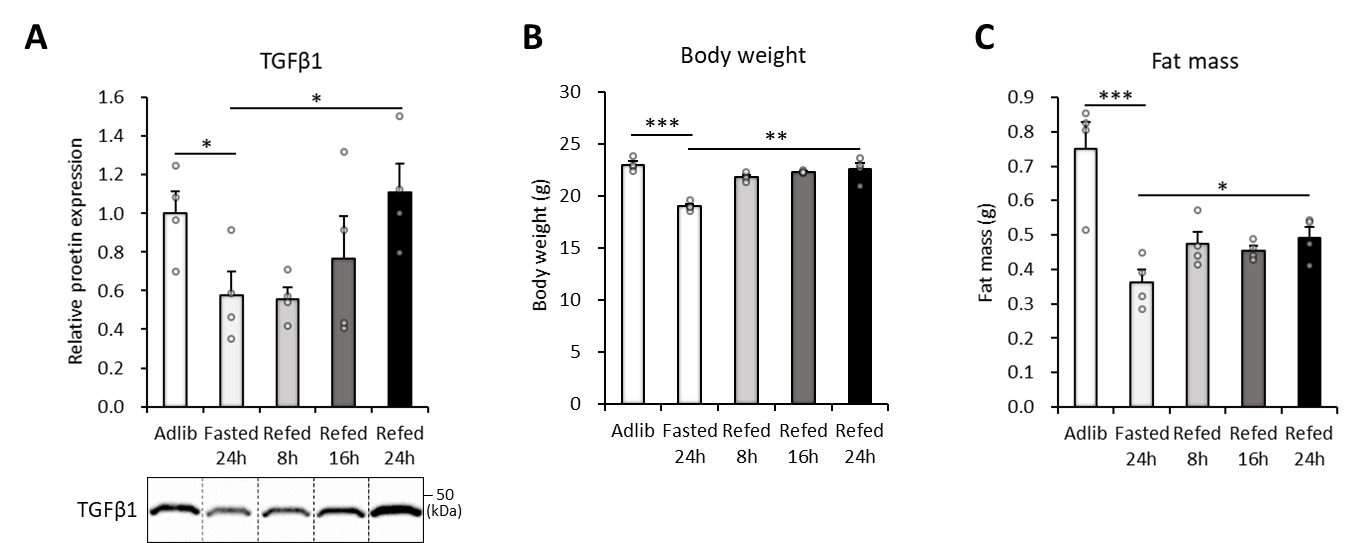


**Supplemental Figure 2.** A-C: TGFβ1 protein expression in WAT (gonadal) (A), body weight (B), and fat weight (gonadal and inguinal WAT) (C) of C57BL6 / J mice fasted for 24 hours and re-fed for indicated time points (n=4).

**Supplemental Figure 3.**
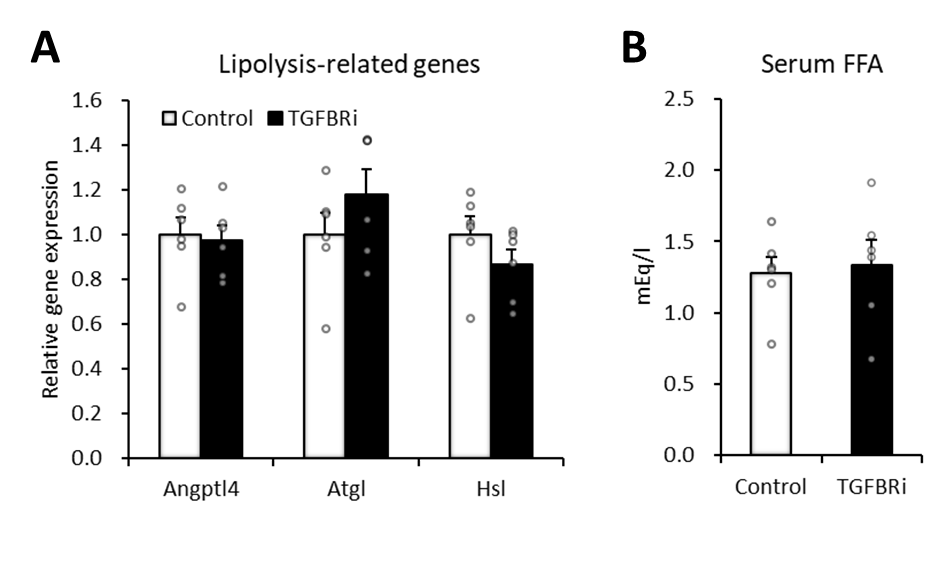
 A and B: Relative gene expression of lipolysis genes in inguinal WAT and serum free fatty acid level of C57BL6/J mice injected 100mg/kg SB431542 under 24 hour fasting condition (n=6). Data are presented as the mean ± SEM.

**Supplemental Figure 4.**
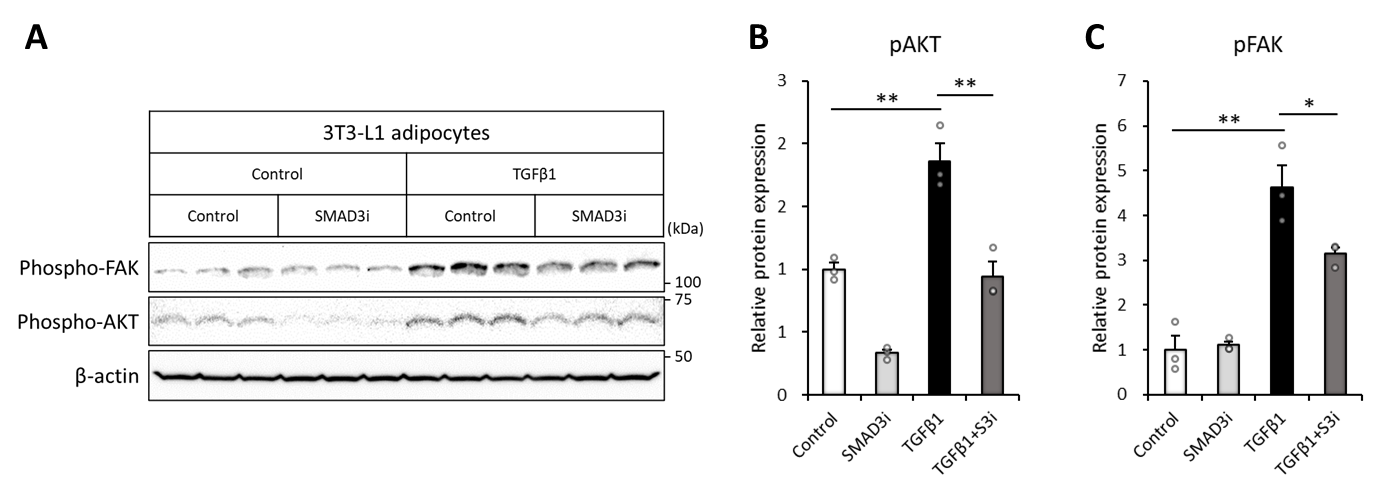
 A-C: Western blot image (A) and relative protein expression of AKT (B) and FAK (C) phosphorylation in 3T3-L1 adipocytes treated with TGFβ1 with/without SMAD3 inhibitor for 12 hours (SIS3 HCl: 10 μM) (n=3). Data are presented as the mean ± SEM. *P<0.05; **P<0.01


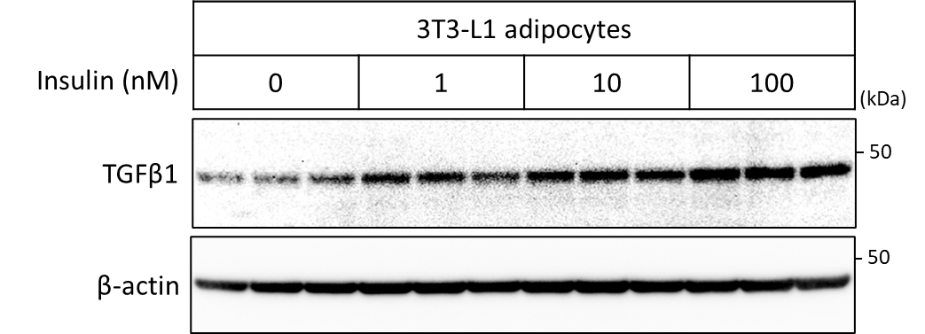


**Supplemental Figure 5.** Western blot image of TGFβ1 protein after 24 hours of insulin treatment in 3T3-L1 adipocytes.

**
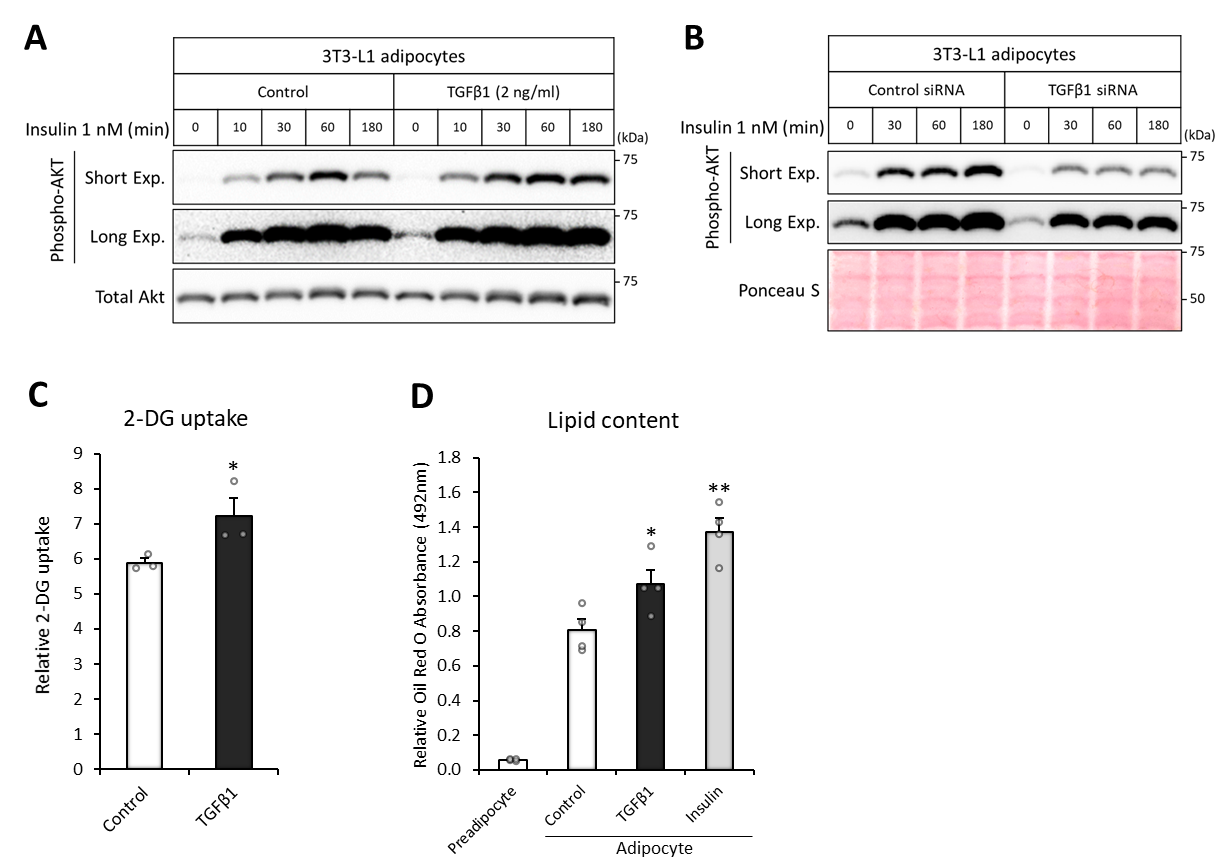
**

**Supplemental Figure 6.** A and B: AKT phosphorylation in 3T3-L1 adipocytes pretreated with TGFβ1 (2 ng/ml) for 3 hours (A) or TGFβ1 siRNA (B) followed by insulin treatment (1 nM) for indicated time points. C: 2-DG uptake in 3T3L1 adipocytes pretreated with TGFβ1(2 ng/ml) for 5h followed by 2-DG treatment for the assay of glucose uptake. D: Absorbance of Oil red O staining in 3T3-L1 adipocytes treated with TGFβ1 (2 ng/ml) or insulin (100 nM) for 48h. Data are presented as the mean ± SEM. *P<0.05; **P<0.01
